# Supplementary material for: A simple silicone elastomer colonization model highlights complexities of Candida albicans and Staphylococcus aureus interactions in biofilm formation
Source: J Med Microbiol. 2025 Jul 14;74(7):002047. doi: 10.1099/jmm.0.002047 (PMC12282317; doi:10.1099/jmm.0.002047)
Supplement: Uncited Supplementary Material 1. [file jmm-74-02047-s001.pdf]

Supplemental table 1: percentage biofilm coverage of field of view.

		Percentage FOV coverage			
		Yeast form		Hyphal form	
		no agar	agar	no agar	agar
Replicate	1	72.20	7.36	10.58	83.61
	2	22.46	61.30	2.50	85.04
	3	39.54	40.38	25.34	85.79
	Average	44.73	36.35	12.80	84.81
	St Dev	25.27	27.19	11.58	1.11

P values	**No agar/agar**		**Yeast form/Hyphal form**	
	Yeast form	Hyphal form	No agar	Agar
	0.404	0.004	0.083	0.043

Supplemental table 2: Statistical analysis of corrected total biofilm fluorescence (CTBF)

525 nm CTBF (*C. albicans* adhered cell density)

		Yeast form		Hyphal form	
		No Agar	Agar	No Agar	Agar
Replicate	1	198969757.8	84532707.71	24228936.32	111993183.6
	2	85535707.33	52965161.77	4305945.118	136709488.1
	3	93217179.62	28534970.97	2549787.703	161269712
	4	46100233.61	33388580.87	36881133.5	332505436
Average		105955719.6	49855355.33	16991450.66	185619454.9
St dev		65354259.74	25415894.73	16507214.2	99969018.72

P values	No agar/agar		Yeast/Hyphal	
	Yeast	Hyphal	No agar	Agar
	0.043	0.016	0.039	0.052

635 nm CTBF (*S. aureus* adhered cell density)

		Yeast form		Hyphal form	
		No Agar	Agar	No Agar	Agar
Replicate	1	103796872.2	45652371.7	2306005.298	14695719.8
	2	9971966.296	29932619.93	3558310.262	22116196.87
	3	8975759.092	4818796.164	28282818.81	99627998.23
	4	27618472.06	5724263.204	12767040.56	190516554.5
Average		37590767.43	21532012.75	11728543.73	81739117.34
St dev		44960396.9	19845893.54	11981366.71	82061466.16

P values	No agar/agar		Yeast/Hyphal	
	Yeast	Hyphal	No agar	Agar
	0.200	0.082	0.198	0.156

Combined CTBF (sum of *C. albicans* and *S. aureus* adhered cell density)

		Yeast form		Hyphal form	
		No Agar	Agar	No Agar	Agar
Replicate	1	302766630	130185079.4	26534941.62	126688903.4
	2	95507673.62	82897781.7	7864255.38	158825685
	3	102192938.7	33353767.14	30832606.51	260897710.2
	4	73718705.67	38218402.28	49648174.06	523021990.5
Average		143546487	71163757.63	28719994.39	267358572.3
St dev		106840717.2	45226082.32	17148256.86	179789571

P values	No agar/agar		Yeast/Hyphal	
	Yeast	Hyphal	No Agar	Agar
	0.066	0.032	0.065	0.083

Supplemental table 3: Statistical analysis of crystal violet staining

Absorbance at 570nm

		Yeast form		Hyphal form	
		No Agar	Agar	No Agar	Agar
**Replicate**	1	0.040	0.047	0.013	0.096
	2	0.054	0.033	0.017	0.082
	3	0.077	0.074	0.013	0.087
Average		0.057	0.051	0.014	0.088
St dev		0.019	0.021	0.002	0.007

P values	**No agar/agar**		**Yeast/Hyphal**	
	Yeast	Hyphal	No Agar	Agar
	0.289	0.002	0.031	0.045

A

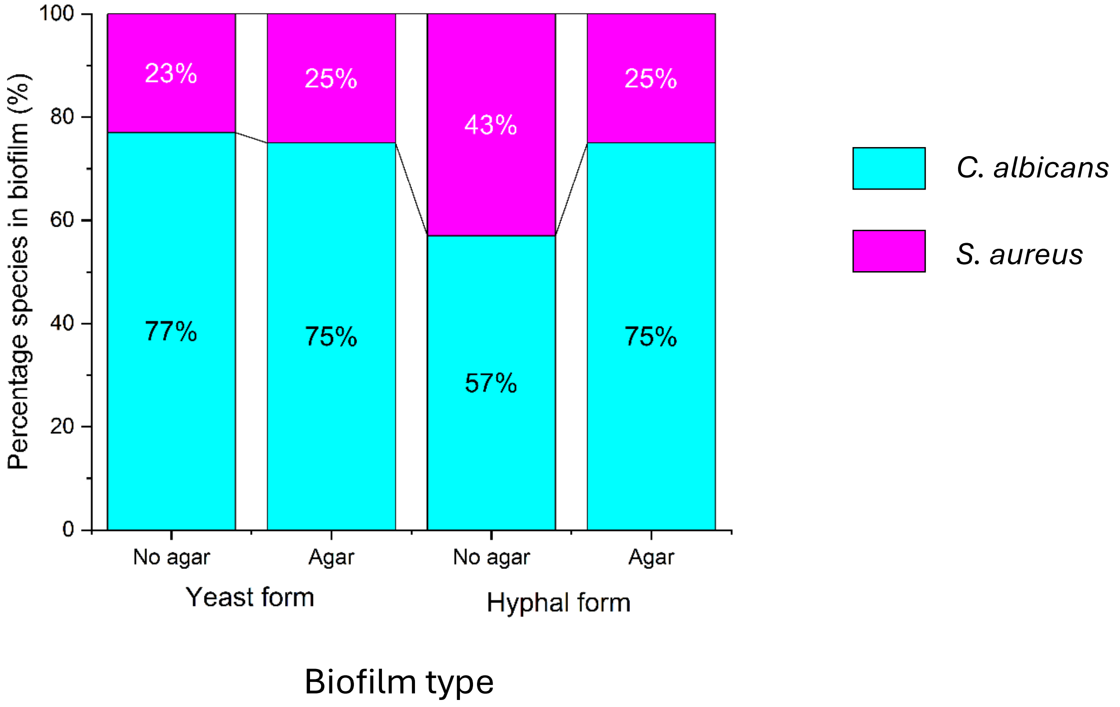

B

		% Species in Biofilm		
		C. albicans	*S. aureus*	Mean deviation
Yeast form	No agar	77	23	± 13
	Agar	75	25	± 11
Hyphal form	No agar	57	43	± 26
	Agar	75	25	± 12

Supplemental Figure 1: Species percentages within biofilms. A: Graph depicting average percentages of species populations as determined by corrected total biofilm fluorescence data from Yeast form and Hyphal form biofilms. **B:** Table of percentages with average deviation from the mean.
